# Childhood Trauma and PTSD Symptoms Increase the Risk of Cognitive Impairment in a Sample of Former Indentured Child Laborers in Old Age

**DOI:** 10.1371/journal.pone.0057826

**Published:** 2013-02-26

**Authors:** Andrea Burri, Andreas Maercker, Sandy Krammer, Keti Simmen-Janevska

**Affiliations:** Department of Psychology, University of Zurich, Zurich, Switzerland; Max Planck Institute of Psychiatry, Germany

## Abstract

A growing body of evidence suggests a link between early childhood trauma, post-traumatic stress disorder (PTSD) and higher risk for dementia in old age. The aim of the present study was to investigate the association between childhood trauma exposure, PTSD and neurocognitive function in a unique cohort of former indentured Swiss child laborers in their late adulthood. To the best of our knowledge this is the first study ever conducted on former indentured child laborers and the first to investigate the relationship between childhood versus adulthood trauma and cognitive function. According to PTSD symptoms and whether they experienced childhood trauma (CT) or adulthood trauma (AT), participants (n = 96) were categorized as belonging to one of four groups: CT/PTSD+, CT/PTSD-, AT/PTSD+, AT/PTSD-. Information on cognitive function was assessed using the Structured Interview for Diagnosis of Dementia of Alzheimer Type, Multi-infarct Dementia and Dementia of other Etiology according to ICD-10 and DSM-III-R, the Mini-Mental State Examination, and a vocabulary test. Depressive symptoms were investigated as a potential mediator for neurocognitive functioning. Individuals screening positively for PTSD symptoms performed worse on all cognitive tasks compared to healthy individuals, independent of whether they reported childhood or adulthood adversity. When controlling for depressive symptoms, the relationship between PTSD symptoms and poor cognitive function became stronger. Overall, results tentatively indicate that PTSD is accompanied by cognitive deficits which appear to be independent of earlier childhood adversity. Our findings suggest that cognitive deficits in old age may be partly a consequence of PTSD or at least be aggravated by it. However, several study limitations need to considered. Consideration of cognitive deficits when treating PTSD patients and victims of lifespan trauma (even without a diagnosis of a psychiatric condition) is crucial. Furthermore, early intervention may prevent long-term deficits in memory function and development of dementia in adulthood.

## Introduction

Early childhood adversity such as physical and sexual abuse, emotional neglect, parental loss, etc., are major risk factors for the development of a range of psychiatric disorders in adulthood, including posttraumatic stress disorder (PTSD) [1], [2]. PTSD occurs following exposure to a traumatic event and is defined by distinct symptom clusters of re-experiencing, avoidance and numbing, and arousal persisting for more than 1 month after trauma [3]. PTSD can have severe long-term consequences and individuals who develop PTSD have an increased risk of major depression, substance dependence, and other health conditions, as well as impaired role functioning and reduced life course opportunities [4], [5].

Recently, links between trauma, PTSD and increased risk of dementia have been suggested. According to several pieces of evidence from animal and human studies, stress experienced early in life induces structural, functional, and epigenetic changes in brain regions involved in cognition, predominantly in the frontal and temporal lobes and the hippocampus [6]. Animal studies demonstrated that early life stress-induced increase of glucorticoids significantly influenced the degree of cognitive impairment with age [7], which is in accordance with the glucocorticoid-cascade-hypothesis of aging [8]. The latter postulates that chronic stress can lead to an increase of cortisol-release which can cause hippocampal atrophy (central region for learning and memory processing). Similarly, several previous study findings have shown that mood disorders such as depression may be associated with a distinct pattern of cognitive impairment [9].

In humans, research has shown that around 70% of individuals suffering from dementia report at least one severe traumatic event before the onset of the disease, as reported by a study conducted at an Dementia Outpatient Clinic in Greece (n = 1′271) [10]. In the like way, in a seminal retrospective cohort study including n = 181′093 predominantly male US war veterans, those who had suffered a PTSD (n = 53,155) had a two-fold increased risk of developing dementia compared with their counterparts without PTSD (n = 127′938) [11]. In addition, PTSD did not appear to be associated with a particular dementia type but rather had an ‘across-the-board effect’ for all dementias, including vascular dementia and Alzheimer's disease. These findings were supported in another recent group comparison study conducted by Qureshi and colleagues, using n = 10′481 US war veterans recruited through the Veterans Integrated Service Network 16 [12]. Veterans aged 65 and older with a diagnosis of PTSD or who were recipients of a Purple Heart (PH) and a comparison group of the same age with no PTSD diagnosis or PH, were divided into four groups and prevalence of dementia was compared across these groups. Results indicated higher incidence and prevalence of dementia in veterans with PTSD compared to veterans without PTSD [12]. Although these findings have important implications for preventive care, it remains to be investigated whether this association is due to a common risk factor underlying PTSD and dementia or to PTSD being a risk factor for dementia.

Overall, current literature suggests that PTSD is associated with cognitive impairment, and a greater incidence and prevalence of dementia. However, whether PTSD-related cognitive changes represent an early marker of dementia or whether they act as risk factors for later dementia needs to be further investigated. Whilst clear associations between adult trauma and cognitive impairment have been repeatedly found, only few studies have measured the long-term consequences of childhood trauma on cognitive function and PTSD in elder individuals. Although such an effect is very likely given that modifiable or stable biographical, psychological, genetic, individual and social factors are causal for the development of dementia, literature reporting on this topic is fairly inconsistent [13]. For example, in a recent study, Majer and colleagues investigated a group of healthy adults with significant exposure to early-life trauma and concluded that physical neglect and emotional abuse might be associated with long-term and working memory deficits in adulthood [14]. Since the authors did not include individuals suffering from PTSD or other trauma-related psychiatric disorders, their study does neither allow to draw conclusions on the association between these disorders and cognitive dysfunction nor does it provide information on the influence of the time-point of traumatization ( i.e. adulthood, childhood) on the extent of cognitive symptoms. Moreover, their restricted sample size (n = 47) might have introduced a bias; therefore replication in bigger samples is needed. Two other studies investigating intelligence, memory and learning deficits in groups of trauma-exposed and non-exposed children and adolescents found no association between cognitive performance and traumatic events in early life [15], [16]. Saigh et al. compared the IQ scores of traumatized youth with PTSD to scores of trauma-exposed and non-exposed comparison groups without PTSD whilst controlling for other major childhood psychiatric disorders. The PTSD group consisting of n = 228 individuals scored significantly lower on the verbal, but not on the performance subtests compared to the n = 276 controls [15]. Furthermore, the scores of the trauma-exposed non-PTSD individuals and non-trauma exposed controls were not significantly different indicating that PTSD and not a history of trauma exposure (without PTSD) is associated with lower verbal IQ. Similarly, Yasik and colleagues found youth with PTSD (n = 29) to have significantly lower scores in verbal memory indices compared with non-traumatized control subjects (n = 40) but no significant differences for general memory or visual memory[16]. The studies expand on the literature documenting memory impairments among adults with PTSD. However, additional research is needed to explore the relation between trauma exposure, diagnostic status, and cognitive performance across a broader range of neuropsychological indices.

To further investigate the role of PTSD as a potential mediator or moderator variable in the association between trauma and cognitive impairment and to determine the influence of PTSD resilience, it is therefore crucial to not only compare cognitive function in individuals with and without prior traumatic experience, but also to look at individuals who developed a PTSD and those who did not. Given also the limited information and inconsistency of current literature, more research is needed to explore the links between cognitive performance and traumatic events early in life. It is likewise possible that the effects of childhood trauma on cognitive function differ in strength from traumas experienced in adulthood and therefore need to be analyzed separately.

The aim of this study was to investigate the association between childhood trauma exposure, PTSD symptoms and cognitive function in a sample of elder adults. To the best of our knowledge no other studies have investigated and compared levels of cognitive function of cohorts of individuals having been exposed to childhood trauma to cohorts of individuals with adulthood trauma. According to previous studies we hypothesized that exposure to childhood trauma, as well as PTSD symptoms would be significantly associated with poorer cognitive function, especially in memory-related domains. To test these hypotheses, childhood trauma exposure, PTSD status, and neurocognitive function was assessed in a unique sample of former Swiss indentured child laborers.

## Materials and Methods

### Sample

The study sample consisted of a subsample (n = 96) of the Swiss ‘Verdingkind’-cohort for which data was available. ‘Verdingkinder’ were indentured child laborers who during their childhood were removed from their usually poor family environment (e.g. parents) by the authorities and sent to work on farms. This was a common feature of Swiss life until the mid-1950's and a dark chapter of Swiss history. Historic studies have shown that many of these children were regularly beaten, and emotionally and sexually abused [17]. In the present study, the following inclusion criteria applied: (Swiss)-German speaking; a minimum age of 60 years; at least one experienced period of indentured child labor; voluntary participation. 57.3% (n = 55) of the participants were male. The mean age of the overall sample was 77.5 years, ranging from 60 to 95 years (Table 1, upper part). The project started in May 2010. Participants were recruited via advertisements in local and national newspapers and magazines, and via specific indentured child laborers' societies and associations. Also, individuals who had previously been mentioned by name in publications or who had talked publicly about their child labor experiences were contacted directly by the research team. All participants provided written consent stating their willingness to participate in this study.

**Table 1:** Sample characteristics of the overall sample and by gender. T-tests were used for the comparison of continuous variables, two-sample tests of proportion for the comparison of categorical/binary variables.

	Overall (n = 96)			Men (n = 55, 57.3%)			Women (n = 41, 42.7%)			
	Mean	SD	Range	Mean	SD	Range	Mean	SD	Range	*p*
Age	77.6	6.3	60–95	77.7	5.3	69–90	77.5	7.5	60–95	ns
GDS	3.8	3.5	0–13	3.5	3.4	0–13	4.3	3.7	0–13	ns
MMS	26.9	2.9	4–30	26.9	3.3	14–30	26.8	2.6	20–30	ns
SISCO	46.2	6.5	22–55	46.8	6.8	22–55	45.5	5.9	34–54	ns
MHV	27.9	8.5	0–40	28.9	8.9	0–40	26.7	7.9	4–38	ns
	**N**	**%**		**N**	**%**		**N**	**%**		***p***
PTSD diagnosis	22	22.9		13	23.6		9	21.9		ns
Childhood trauma	53	56.4		29	53.7		24	60.0		ns
Physical abuse	17	21.3		14	25.9		6	15.0		*
Sexual abuse	11	14.9		3	5.6		11	27.5		*
Emotional abuse	11	11.7		4	7.4		7	17.5		ns
Self-experienced danger of death	2	7.5		6	11.1		1	2.5		ns
Alcohol addiction or abuse	2	2.1		2	3.6		–	–		ns
Substance addiction or abuse	1	1.0		–	–		1	2.4		ns

Note: * = p<.05; ns  =  non-significant

GDS  =  Geriatric Depression Scale; MMS  =  Mini Mental State Score; Sisco  =  Sidam score; MHV  =  Mill Hill Vocabulary Scale

### Ethics Statement

The study was conducted following the ethical standards of the German and Swiss psychological associations. According to article §29.2–4 of the ‘Patient law of the Canton Zurich, 5.4.2004’, formal approval of the project was not necessary as no patients were recruited and strict standards of voluntariness, confidentiality and respondent protection were observed.

### Material

Socio-demographic information was assessed with self-constructed questions.

### Childhood adversity and traumatic life experiences

As part of the clinical interview, the Composite International Diagnostic Interview (CIDI) trauma events list [18], [19] served as a guide to enquire the most severe traumatizing life experience participants had gone through. Additionally, the verbal descriptions of their most severe traumatic events were assessed and categorized into childhood (CT) or adulthood traumatic (AT) experience based on when the traumatizing event had occurred. Typically, periods of indentured laboring ended at the age of 18, therefore this is the cut-off chosen to distinguish between CT and AT. CT were further sub-classified into sexual abuse, emotional abuse/neglect, physical abuse, and self-experienced death threat, severe accident/illness, witnessing a severe accident, losing a close member of the family or reference person, and other/not specified (e.g. imprisonment). Apart from those subclasses AT further included: severe accident/illness, witnessing a severe accident, losing a close member of the family or reference person, and other/not specified (e.g. imprisonment).

In individuals reporting childhood adversity the most frequent type of trauma was physical violence (32.1%), followed by sexual abuse (20.8%) and emotional abuse/neglect (20.8). The most frequently reported traumas in adulthood were ‘other’ (25.6%), witnessing death/accident (20.9%) and death of a family member (16.3%). Only 6.9% reported physical or sexual violence and none of the individuals reported emotional abuse/neglect as their most severe traumatic event.

#### Symptoms of post-traumatic stress disorder (PTSD)

The 7-item short screening scale for PTSD (SSS) is an empirically derived instrument used to discriminate individuals with a diagnosis of PTSD from healthy ones [20]. It was designed after the DSM-IV criteria for PTSD by an iterative series of sensitivity and specificity analyses and thus constitutes a short form of the Posttraumatic Diagnostic Scale (PDS) [21], [22]. Five of the seven symptoms relate to the avoidance and numbing symptom cluster and two to the hyper-arousal symptom cluster. Respondents rate each item as either ‘yes’ or ‘no’ and the overall score is computed by adding the number of ‘yes’ responses. The authors of the SSS suggest a cut-off score of 4 which best balances the scale's sensitivity (80%) - the ability to detect patients with PTSD -, and specificity (97%) - the ability to detect patients who do not have PTSD. A German version of the scale is available [23]. The psychometric properties of the PDS from which the SSS was derived has been extensively investigated and been considered good to very good [21]. The specific performance characteristics of the SSS (including the German version) have recently been investigated in two studies [23], [24] and have been considered very good.

#### Assessment of Cognitive Function

Cognitive function was assessed with the Structured Interview for Diagnosis of Dementia of Alzheimer Type, Multi-infarct Dementia and Dementia of other Etiology according to ICD-10 and DSM-III-R (SIDAM) [25]. The SIDAM comprises a test performance part, a section for clinical judgment and third party information to determine psychosocial impairment. The SIDAM test performance part consists of a range of cognitive tests that constitute a short neuropsychological battery with 55 questions, including all 30 items of the Mini-Mental State Examination (see below) [26]. Within the context of this study, only the performance part was conducted. It yields a maximum score of 55 (*SI*DAM *sco*re or SISCO). The SISCO can be subdivided into several cognitive domains including: orientation, immediate recall, delayed recall, long term memory, intellectual abilities, verbal abilities/calculation, visuo-spatial function, and aphasia/apraxia. The composite subscore ‘memory’ is derived by summing the performance in the subdomains ‘immediate recall’ (of numbers and words), ‘delayed recall’ (of verbal and figural material) and ‘long term memory’ (biographical memory).

The SIDAM has a high overall test-retest reliability which equally holds true on the diagnostic, criterion and item level. It separates reliably between subjects with DSM-III-R and ICD-10 dementia from those without such a disorder. Furthermore, good congruence was found between SIDAM diagnosis and corresponding ICD-9 expert diagnosis [25]. Age- and education-specifics norms for the assessment of dementia have been obtained in a German population-based sample of n = 1001 individuals aged >75 years [26]. Independent of education, a Sisco score of 36 for individuals >80 years, and a score of 38 for individuals aged 75 to 79 years were found to differentiate best (i.e. best specificity-sensitivity profile) between clinically demented and healthy individuals.

In the current study reliability coefficients for the various subscale of the SIDAM ranged from α = 0.51 (for orientation) to α = 0.74 (for short term memory).

The Mini-Mental State Exam (MMSE) is one of the most widely used screening measures of general cognitive ability [27]. The brief 30-point questionnaire is used to screen for levels of cognitive function, with items assessing six different domains including: cognition, including orientation, word recall, registration of new information, attention and calculation, language abilities, and visuo-spatial ability. Scores on the MMSE range from 0 to 30, with scores of 25 or higher being considered normal. The instrument has been used within different cultural and ethnic groups and translated into many languages. Good psychometric properties (reliability and validity) have repeatedly been demonstrated [27].

The ‘Vocabulary Test’ is the German version of the Mill Hill Vocabulary Scale (MHV) used for the measurement of acquired verbal knowledge [28], [29]. The MHV represents the verbal test part of the Raven's Progressive Matrices. Contrary to most other vocabulary tests which assess passive vocabulary or recognition performance, the MHV allows the assessment of active vocabulary. The test consists of a list of words divided into two parallel lists of 44 words (set A and B) and participants are asked to explain the meanings of the words. Alternatively, participants can chose a synonym from a selection of six words. The psychometric properties of the test have been extensively investigated and considered good to excellent, with internal consistencies are .93 and .91, respectively (for definition vs. synonym picking task) and good convergent and discriminant validity [29].

#### Depression

The Geriatric Depression Scale (GDS) is a commonly applied self-report instrument used to specifically identify depression in older people [30]. A short version of the original questionnaire (GDS-SF) containing 15 questions is available and was used within the context of this study. The short version has been found to be an adequate substitute for the original 30-item scale. The questions are answered ‘yes’ or ‘no’, instead of a five-category response set. This simplicity enables the scale to be used with ill or moderately cognitively impaired individuals. Scores greater than 3 suggest the presence of depression. The test sensitivity ranges from 79% to 100%, specificity from 67% to 80%. Validation of the German version of the GDS found the instrument to be a reliable and valid screening instrument with an average item discrimination of 0.5, an average item difficulty of *P* = 43, low inter-item correlation *r* = 0.2 and a very high internal consistency (Cronbach's α = 0.9) [31]. In the current study GDS showed adequate internal consistency reliability (Cronbach's α = 0.8).

### Design

According to the information on PTSD symptoms (derived from the SSS) and whether participants experienced CT (including either sexual abuse, emotional abuse, physical abuse or near to death experiences that happened during the indentured child laboring period) or AT (additionally including near to death experiences, severe accident/illness, witnessing a severe accident, losing a close member of the family or reference person, and other/not specified) individuals were categorized as belonging to one of four groups: with CT and a PTSD (CT/PTSD+), with CT and no PTSD (CT/PTSD-), with AT and PTSD (AT/PTSD+), with AT and no PTSD (AT/PTSD-). Note that in all subsequent paragraphs PTSD+ does not refer to a clinical diagnosis of PTSD but solely describes traumatized individuals who screened positively for PTSD symptoms according to the SSS.

### Statistical Analyses

For descriptive statistics, two-sample tests of proportions to assess differences between gender on categorical and binary data were used. Student's t-tests were applied to assess differences between gender means on continuous variables. We used point-biserial correlation coefficient and logistic regression to examine the relationship between the binary variables representing PTSD symptoms and childhood trauma status and the various continuous variables measuring cognitive function. Because normality of the data could not be assumed, given the low sample size in some of the groups, univariate Kruskal-Wallis analyses (non-parametric test, equivalent to ANOVA) were calculated to compare the score differences between the groups for the continuous cognitive function variables. Bonferroni corrected, multiple sample contrasts were performed post-hoc to these analyses. To control for the influence of depression as a potential covariate on the cognitive function outcome variables, Kruskal-Wallis tests were performed on covariate-adjusted (i.e. GDS) residuals. Covariate-adjusted residuals were obtained from the overall regression line fit to the entire data set.

All tests were two-tailed. For all analyses, a *P* value less than0.05% or odds ratios (OR) with a 95% confidence interval (CI) not including ‘1’ were considered statistically significant, unless stated otherwise. Data handling and analyses were undertaken using STATA (Version 10.0, 2008, StataCorp, College Station, TX, USA).

## Results

According to the SSS (cut-off score of 4), 22 individuals (22.9%) screened positively for PTSD symptoms. Thus, the 96 participants were assigned to the four groups as follows: n = 10 in the CT/PTSD+ group, n = 31 in the CT/PTSD- group, n = 12 in the AT/PTSD+ group, n = 43 in the AT/PTSD- group.

Participants further reported a high prevalence of depressive symptoms, with an average geriatric depression score of 3.8 (SD = 3.5; Table 1). Overall, participants showed mild to no cognitive impairment, with average MMS and SISCO scores of 26.9 (SD = 2.9) and 46.2 (SD = 6.45), respectively.

In terms of cognitive function variables and prevalence of PTSD symptoms, no significant gender differences could be detected (Table 1). Male and female participants, however, differed significantly in prevalence of sexual and physical abuse, with male more frequently reporting previous physical abuse (25.9% vs. 15.0%, *P*<0.01) and females more frequently sexual abuse (5.6% vs. 27.5%, *P*<0.01).

The main analysis compared the four groups of childhood/adulthood trauma (CT/AT) and PTSD (+/−) for indicators of cognitive decline or dementia processes (Table 2). The mean age across all four groups was similar. Furthermore, the four groups did not differ significantly in terms of depressive symptoms. As expected there was a statistically significant difference between groups in levels of cognitive function as measured by the MMS and SISCO score (*X^2^* = 9.3, *P*<0.05 and *X^2^* = 10.1, *P*<0.05, respectively). Upon investigation of the specific cognitive sub-domains, significant group differences were detected for higher cortical function (*X^2^* = 12.3, *P*<0.001), verbal numeracy (*X^2^* = 8.8, *P*<0.05) and construction skills (*X^2^* = 6.3, *P*<0.05). The group differences in MHV scores fell short for significance (*X^2^* = 7.5, *P*<0.06). Overall, analyses showed that participants screening positively for PTSD symptoms were more likely to report poorer cognitive function compared to individuals without PTSD symptoms in both the main score and the three sub-domain scores.

**Table 2:** Results of analysis of variance (Kruskal-Wallis test) for the means of the cognitive function variables across the four CT/PTSD groups.

	CT/PTSD +(n = 10)		CT/PTSD-(n = 31)		AT/PTSD+(n = 12)		AT/PTSD-(n = 43)			
	Mean	SD	Mean	SD	Mean	SD	Mean	SD	*X^2^*	Contrasts
Age	75.9	4.5	75.9	6.7	78.5	7.1	78.9	5.9	5.5	--
GDS	4.6	3.9	3.8	3.4	4.3	3.7	3.5	3.6	1.3	--
MMS	25.6	3.4	26.9	3.2	25.5	2.8	27.6	2.6	9.3*	1<4, 3<4
SISCO	42.3	6.4	46.7	6.7	43.0	6.5	47.8	5.7	10.1*	1<4; 3<4
*Orientation*	9.5	0.7	9.5	0.9	9.8	0.4	9.7	0.6	1.6	--
*Memory total score*	13.1	2.9	15.6	3.5	14.2	3.4	15.5	3.6	6.6	--
*Memory immediate reproduction*	4.1	0.3	4.4	0.6	3.8	1.1	4.3	0.6	3.1	--
*Short term memory*	4.6	2.1	5.7	2.4	4.8	2.1	5.6	2.3	4.4	--
*Long term memory*	4.4	1.8	5.5	1.4	5.6	0.9	5.5	1.3	3.8	--
*Intellectual performance*	4.5	0.9	4.9	0.6	4.3	1.5	4.7	0.7	1.3	--
*Higher cortical function*	15.2	3.1	16.6	2.9	14.7	2.4	17.4	2.6	**12.3** **	3<4
*Verbal numeracy*	4.1	2.2	5.5	1.6	4.6	1.6	5.7	1.4	8.8*	3<4
*Construction skills*	1.4	1.0	1.7	1.1	1.0	0.7	1.9	1.2	6.3*	3<4
*Aphasia aparaxia*	9.5	0.8	9.3	0.9	9.1	1.3	9.6	0.8	2.0	--
MHV	26.1	6.6	30.3	6.3	21.6	10.8	28.5	8.8	**7.5^†^**	--

Note: ^†^ = p<.06, * = p<.01, ** = p<.001

GDS  =  Geriatric Depression Scale; MMS  =  Mini Mental State Score; Sisco  =  Sidam score; MHV  =  Mill Hill Vocabulary Scale

To further investigate between which groups the differences in scores were significant we performed multiple sample contrasts. Post-hoc analyses indicated that individuals screening positively for PTSD symptoms reported poorer cognitive function, with significantly lower MMS and SISCO scores, compared to individuals without PTSD symptoms (Table 2). Especially individuals with PTSD symptoms and reporting CT showed poorer cognitive function. Similarly, these groups also differed significantly with respect to higher cortical function, verbal numeracy and construction skills.

Because previous literature has shown a moderating role of depressive symptoms on cognitive functioning, we included GDS scores as a confounder in the analyses of all available variables. Even after controlling for depression, the difference between the groups remained significant for the MMS and the Sisco score (*X^2^* = 9.9, *P*<0.05 and *X^2^* = 12.5, *P*<0.001, respectively), as well as for the sub-domains of higher cortical function and verbal numeracy (*X^2^* = 12.9, *P*<0.001 and *X^2^* = 8.2, *P*<0.05, respectively). Contrary to the initial results, the significant group difference for construction skills could not be detected when controlling for depression.

## Discussion

Early-life trauma is a major risk factor and elicitor for the development of PTSD, a condition which cardinal features are changes in cognitive function. So far, only a handful of studies have measured the long-term consequences of childhood trauma on cognitive function in healthy adults.

This is the first study ever conducted on former indentured Swiss child laborers and the first to investigate the relationship between childhood versus adulthood trauma levels of cognitive function in later life. In this present study we found associations between childhood trauma exposure, PTSD symptoms and cognitive performance. In particular, individuals who screened positively for PTSD symptoms showed lower levels of cognitive function in the SIDAM total score (SISCO), the MMS score and the specific cognitive domains of higher cortical function, construction skills and verbal numeracy compared to individuals without PTSD symptoms. As to be expected, cognitive function was consistently highest across all domains in individuals without reported childhood trauma and without PTSD symptoms. Furthermore, individuals with adulthood trauma and screening positively for PTSD symptoms were at a similarly high risk for poorer cognitive performance compared to individuals with childhood trauma and screening positively for PTSD symptoms. Finally, we found that all associations between PTSD symptoms and cognitive function - apart from construction skills - were moderated by depressive symptoms. In other words, accounting for depression led to stronger associations between PTSD symptoms and lower levels of cognitive function. No differences in any of the variables studied could be found between male and female individuals.

Our results add to a growing body of literature supporting a relationship between childhood trauma exposure, PTSD and the development of cognitive dysfunction in elder individuals. Qureshi et al. (2010) for example found a somewhat similar picture in an analogous group comparison design including individuals with ICD-9 diagnoses of dementia [12]. In their study, almost two-fold higher dementia prevalences were reported in the two PTSD groups compared to the non-PTSD groups. Additionally, their group most similar to our ‘AT/PTSD+’ group showed the highest prevalence of a dementia diagnosis. Similarly, the results of the most comprehensive Yaffe et al (2010) study indicated an association between PTSD and increased dementia risk [11]. The study used war veterans who usually combat at ages around 18–28 years, therefore relying on traumatization in early adulthood. Together with our findings this tentatively indicates that adulthood trauma-related PTSD may outweigh childhood-related PTSD in its relevance as a causal agent for cognitive decline. This assumption is further supported by a recent study on childhood Holocaust survivors conducted by the research group around Ravona-Springer who found no evidence for an increased risk for dementia at old age [32]. Although overall these preliminary findings cautiously point towards a more substantial influence of traumatic effects experienced in adulthood on cognitive decline, they need to be replicated and supported by other studies. In future it will be especially important to diligently assess the amount and severity of traumatic childhood and adulthood experiences. At this stage it is not possible to exclude the existence of cumulative or masking effects of different trauma types and times of occurrence which could be an explanation for the rather inconsistent research outcomes.

Our main analyses were supported and even strengthened when taking depressive symptoms into account (for additional analyses on depression in the current sample, see Kuhlman et al., 2012) [33]. These results are in line with findings from previous studies reporting an independent association between depressive symptoms and cognitive performance [34]. It has been argued that this relationship can be best explained by neurobiological models suggesting a link between depression, hypothalamo-pituitary-adrenal (HPA) axis dysfunction (i.e. depression-related cortisol hypersecretion) and cognitive dysfunction [34], [35].

A similar model has been proposed for symptom development and psychobiologic changes occurring in individuals with PTSD. Most of the post-traumatic symptoms have a biological correlate; this is particularly true for hyperarousal – one of the elementary characteristics of PTSD, defined as a heightened state of psychological and physiological tension. The body responds to increased acute physical or psychological stress by activating the HPA-axis which leads to the release of adreno-corticotrophin (ACTH) from the anterior pituitary and produces an increase of cortisol levels. Over the long term, such hyperarousal and the increased levels of corticosteroid secretion may disrupt somatic, cortical, cognitive and affective processing by having neurotoxic effects [8], [36]. Such hypercortisolemia has previously been associated with increased risk of dementia. It is very likely that PTSD-related or PTSD-symptom related (e.g. hyperarousal) alterations in the HPA-axis produce neuroanatomical changes including volume changes in the frontal lobe, lower neuronal density in the medial temporal lobe and hippocampal atrophy [37]–[39] and thereby evoke significant changes in cognitive functioning.

In another longitudinal validation study conducted by Bickel and colleagues (2007) the subdomains' predictive validities for dementia were explored. The authors found the SIDAM total score and the subscale ‘memory’ (especially ‘short-term memory’) and ‘higher cortical function’ to have the strongest predictive power for the development of later dementia [40]. This is somewhat in line with our findings, where significant group-differences could be detected for the SIDAM total score and the domains of higher cortical function, construction skills and verbal numeracy (as part of the higher cortical function subscale). This provides additional support for our findings of tentative effects of trauma and PTSD symptoms on cognitive function. To further explore whether such changes are static or develop progressively and how they correlate with disease (PTSD) duration, longitudinal designs are needed. In addition, there might be differences in the specific causes leading to cognitive decline in people with PTSD. Recent research, such as the study conducted by Dretsch et al. (2012) on 46 war veterans with PTSD, indicate that cognitive deficits appear to be partially attributed to anxiety and depression symptoms [41]. Although the mediating effects of depression were controlled for in our study, we cannot exclude a potential effect of other mediators (such as e.g. anxiety) on the detected group differences in neuro-cognitive functioning.

### Limitations

Our results should be considered in light of several limitations. First, due to the uniqueness of the indentured child laborers sample, the overall, as well as the group-specific sample sizes were relatively small. This might have led to restricted power in statistical testing. The chi-square approximation of the Kruskal-Wallis test statistics, however, has been proven to be highly satisfactory also when sample sizes are small [42]. Given the singularity of our study sample, the possibility to recruit additional participants is unlikely. Nevertheless, replication of the study in other populations with childhood trauma and PTSD could allow more detailed group-comparison of cognitive function. Second, our sample is specific in terms of life histories or events' characteristics, thus the study might have limited generalizability to other populations and other subpopulations with histories of severe childhood trauma. However, long-lasting traumatic experiences during childhood are prevalent even in more common community samples [43]. Additionally, there might have been selectivity bias among the former child laborers who were willing to participate in a study asking about their traumatic experiences and the ones that were not. Similarly, trauma group studies have demonstrated that traumatized persons who have elevated PTSD-related avoidance symptoms tend to not participate in such studies. Third, we cannot exclude the possibility that our data are affected by biases (such as distortions due to forgetting, nondisclosure, mood states, reporting bias, etc.) given the fact that we relied on retrospective self-reports of childhood experiences. Previous evidence, however, has shown that the available standardized measurement instruments are sufficiently valid to warrant their use in retrospective recall studies [44]. As a fourth limitation, we did not consider effects of adulthood trauma and life stress that might have mediated the relationship between childhood trauma and cognitive decline. It is known that individuals with early trauma more frequently experience adulthood stress, and moreover, are sensitized to the effects of such stressors [45]. Fifth, several important variables that are known to affect cognition - such as duration, severity, and treatment of PTSD, medication use, years of education, and premorbid intelligence - could not be included in the analyses due to the unavailability of such data. Sixth, owing to the cross-sectional design the causal relationship between early adversity and cognitive dysfunction could not be determined. In the context of the second assessment wave, additional information on participants' cognitive status is currently being collected and will allow more specific statements regarding the relationship between earlier trauma and levels of cognitive function and potential long-term impairment. Finally, attention must be paid to the assessment of trauma type and severity, as well as PTSD status. The SSS is limited to measuring PTSD symptoms in persons who have experienced DSM-IV traumatic events, thus ascertainment of an index event in individuals exposed to multiple events is not covered by the scale. Also, the SSS is an efficient method to screen for PTSD in epidemiologic and clinical studies but does not substitute for a psychiatric diagnosis. Likewise, the SIDAM and MMSE solely allow assessment of cognitive function and decline. They do not permit a diagnosis of dementia which requires at least the exclusion of other psychiatric diseases and somatic disorders (such as hypovitaminosis, hypothyreodism, normal pressure hypdrocephalus), as well as multi time-point assessment for the identification of transient versus persistent cognitive impairments (i.e. dementia).

## Conclusion

Findings from this study support the hypothesis that PTSD symptomatology affects cognitive function and may put individuals at risk for later dementia. These findings are consistent with previous reports of neuro-anatomical changes in individuals suffering from PTSD or PTSD symptoms. This elevated risk for dementia seems to be particularly relevant, when trauma appeared in adulthood. Overall, our results suggest that poor cognitive function in late adulthood may be partly a consequence of PTSD symptoms but can also develop as a consequence to traumatization or be aggravated by them. Proposals for future research are given and longitudinal data providing more detailed information on the reported associations and to further investigate potential mediators and moderators is warranted.
